# Muscle Abnormalities and Meat Quality Consequences in Modern Turkey Hybrids

**DOI:** 10.3389/fphys.2020.00554

**Published:** 2020-06-12

**Authors:** Marco Zampiga, Francesca Soglia, Giulia Baldi, Massimiliano Petracci, Gale M. Strasburg, Federico Sirri

**Affiliations:** ^1^Department of Agricultural and Food Sciences, Alma Mater Studiorum – University of Bologna, Bologna, Italy; ^2^Department of Food Science and Human Nutrition, Michigan State University, East Lansing, MI, United States

**Keywords:** turkey, muscle growth, myopathy, muscle abnormality, meat quality

## Abstract

Turkey meat is the second most consumed poultry meat worldwide and represents an economic source of high-quality protein for human consumption. To fulfill the increasing demand for turkey meat, breeding companies have been selecting genetic lines with increased growth potential and breast muscle proportion. Moreover, the progressive shift toward further processed products has emphasized the need for higher standards in poultry meat to improve its technological characteristics and functional properties (i.e., water-holding capacity). However, as observed for broiler chickens, a growing body of scientific evidence suggests that the intense selection for the aforementioned traits could be associated with a greater occurrence of growth-related myopathies and abnormalities and, consequently, to increased downgrading rates and overall reduction of meat quality characteristics. In the past, muscle abnormalities such as deep pectoral myopathy, pale-soft-and-exudative-like meat, and focal myopathy have been reported in turkey lines selected for increased growth rate. In addition, the presence of white striations in the superficial layer of pectoralis major muscle, as well as the tendency of muscle fiber bundles to separate resulting in an altered breast muscle structure, has been detected in commercial turkey abattoirs. Furthermore, past investigations revealed the presence of another quality issue depicted by an overall toughening of the breast muscle. These meat abnormalities seem to macroscopically overlap the white striping, spaghetti meat, and wooden breast conditions observed in pectoral muscle of fast-growing, high-breast-yield chicken hybrids, respectively. Considering the high economic impact of these growth-related abnormalities in broilers, there is an increasing interest of the turkey industry in estimating the occurrence and the impact of these meat quality issues also in the modern turkey lines. Studies have been recently conducted to assess the effect of the genotype on the occurrence of these emerging growth-related defects and to evaluate how meat quality properties are affected by white-striping condition in turkeys, respectively. Therefore, this review aims to provide a critical overview of the current understanding regarding the growth-related abnormalities and their impact on meat quality in modern turkey hybrids with the hope that this information may improve the knowledge concerning their overall effect on poultry meat.

## Introduction

According to recent estimates, poultry meat consumption is increasing worldwide regardless of the country and the income level (OECD/FAO, 2016). The turkey sector represents one of the major pillars of the poultry industry (FAOSTAT, 2020), with turkey meat being the second most consumed poultry meat worldwide with an average annual consumption of 4.0 and 7.3 kg/per capita in the European Union (EU) and the United States, respectively (AVEC, 2019). Consumers, particularly in Western countries, are increasingly attracted toward breast meat, mainly because of its perceived healthy profile and the variety and organoleptic qualities of processed poultry meat products (Petracci and Cavani, 2012; Petracci et al., 2015). Therefore, to fulfill the increasing demand for breast meat, breeding companies have targeted the selection toward genetic lines with rapid breast muscle development and high carcass yield, achieving astonishing results as summarized by Havenstein et al. (2007). In addition, the progressive shift toward further processed products has emphasized the necessity for higher standards in poultry meat to improve its technological characteristics and functional properties (e.g., water-holding capacity) (Barbut, 2015; Petracci et al., 2015).

However, Wilson et al. (1990), finding more fragmented muscle fibers and higher plasma creatine kinase concentration in fast-growing turkey lines compared to slow-growing ones, hypothesized that the selection for growth rate and muscle development has resulted in muscles that have “outgrown their life-support systems,” leading to alterations in muscle morphology, physiological state, and biochemistry. Further studies have been supporting this hypothesis, suggesting that the selection for the aforementioned traits in modern turkey lines could be associated with alterations in the musculoskeletal structure (Sosnicki and Wilson, 1991; Velleman et al., 2003; Hocking, 2014; Velleman, 2015) and a greater occurrence of growth-related abnormalities (Sosnicki and Wilson, 1991; Mahon, 1999; Owens et al., 2009), as well as impaired meat functionality and technological traits (Updike et al., 2005), which will be discussed in the following paragraph.

## Historical Evolution of Meat Abnormalities in Turkeys

The first scientific reports regarding growth-related muscle abnormalities in turkeys were published during the 1960s and 1970s. As previously stated, the extraordinary improvements achieved in growth rate and breast muscle yield were accompanied by alterations of muscle morphology, physiology, and biochemistry with negative implications for overall meat quality traits and downgrading rates. One of the first reported growth-related meat abnormalities in turkeys was the deep pectoral myopathy (DPM). Originally described by Dickinson et al. (1968) as a “degenerative myopathy” affecting the supracoracoideus muscle (pectoralis minor or deep pectoralis muscle), DPM is characterized by necrosis, dry, stringy, woody-like texture, atypical color (i.e., “green muscle disease”), and gross edematous appearance (Sosnicki and Wilson, 1991; Jordan and Pattison, 1998; Table 1). It is widely accepted that DPM is caused by an ischemic condition induced by the swelling of the muscle during exercise (Orr and Riddell, 1977; Siller and Wight, 1978; Siller et al., 1979; Jordan and Pattison, 1998). Indeed, the compartmentalization of the deep pectoral muscle between the inelastic muscle fascia and the keel bone can determine the swelling of the supracoracoideus muscle during exercise (e.g., wing flapping), resulting in the partial occlusion of the cranial and caudal pectoral arteries leading to a limited blood circulation to the muscle, which, in turn, triggers degenerative cellular processes (Siller, 1985; Sosnicki and Wilson, 1991). Although DPM does not represent a public health concern or an animal welfare issue, affected fillets need to be trimmed and subsequently discarded as unacceptable for human consumption. However, the selection against DPM and the adoption of specific management operations have significantly reduced the incidence of this defect (Sosnicki and Wilson, 1991), which now occurs only sporadically in commercial broiler and turkey flocks.

**TABLE 1:** Description of the main breast muscle abnormalities in modern commercial turkeys and their impact on overall meat quality.

Breast muscle abnormality	Macroscopic traits	Causes	Current occurrence level	Impact on meat quality	Main references
Deep pectoral myopathy	Atypical coloration (from reddish to greenish) of *P. minor*	Ischemic condition caused by muscle swelling during exercise	Sporadic	Increased trimming and discarding rate as affected fillets, presenting necrosis, dry, stringy, and hardened texture, atypical color, and gross edematous appearance, are unacceptable for human consumption	Dickinson et al., 1968; Orr and Riddell, 1977; Siller and Wight, 1978; Siller et al., 1979; Siller, 1985; Sosnicki and Wilson, 1991; Jordan and Pattison, 1998
Pale, soft, exudative–like condition	Abnormal light or pale color, soft texture, excessive juice loss	Rapid and/or excessive postmortem muscle acidification due to acute preslaughter stress	High, especially during hot seasons	Downgrading due to muscle abnormal appearance and poor functional properties for further processing; loss of protein functionality, which results in reduced water-binding capacity and cook yield	Barbut, 1993, 1996; Sosnicki, 1993; Sosnicki et al., 1995, 1998; Pietrzak et al., 1997; Owens et al., 2000; Fraqueza et al., 2006; Barbut et al., 2008; Chiang et al., 2008; Malila et al., 2014; Carvalho et al., 2018
Dark-firm-dry condition	Abnormal dark color and dry appearance	Reduced postmortem muscle acidification due to antemortem chronic stress inducing glycogen depletion	Moderate	Downgrading due to muscle abnormal appearance and greater potential spoilage from organisms and food-borne pathogens ascribable to the higher ultimate pH; higher water-binding capacity if used for further processed products	Mallia et al., 2000; Fraqueza et al., 2006; Carvalho et al., 2018; Henrikson et al., 2018
White striping	White striations of variable thickness parallel to muscle fibers direction in the ventral surface of pectoralis major	Alterations of regular muscle architecture during growth with abnormal deposition of adipocytes	Moderate	Slight effect on meat proximate composition (increased fat content) and technological traits	Soglia et al., 2018; Mudalal, 2019; Zampiga et al., 2019
Spaghetti meat-like	Separation of muscle fiber bundles in the superficial layer of the cranial region of pectoralis major	Progressive rarefaction of perimysial connective tissue during growth	Sporadic	Affected parts are trimmed, whereas no information is available about possible meat quality implications	Zampiga et al., 2019
Wooden breast	Hardening of pectoralis major muscle	Connective tissue proliferation	Not yet detected	–	–

Another very well-studied and described meat alteration in turkeys is pale, soft, and exudative (PSE)–like meat (Table 1). PSE–like meat is characterized by abnormally light or pale color, soft texture, and reduced water-holding capacity and cooking yield (Barbut, 1993, 1996; Sosnicki et al., 1998). This condition can be ascribed to antemortem stressors, such as the sudden onset of a heat wave and the transportation of turkeys to slaughter facilities, which induce an accelerated glycolytic metabolism of breast muscle fibers leading to increased muscle temperature and rapid development of rigor mortis (fast-acidifying meat, pH < 6 within 1 h postmortem) (Pietrzak et al., 1997). Alternatively, the breast muscle may reach a low ultimate pH (acidification of a greater extent) without any remarkable alterations in the acidification rate (acid meat, pH < 5.8) (Sosnicki et al., 1998; Barbut et al., 2008). The combination of low pH and high postmortem breast muscle temperature may result in a partial protein denaturation and hence loss of protein functionality (Sosnicki, 1993; Sosnicki et al., 1995). In addition, analysis of gene expression patterns of normal and PSE-like turkey meat suggests that birds yielding PSE-like meat displayed altered expression of proteins associated with calcium homeostasis, signaling pathways regulating actin cytoskeleton structure, carbohydrate metabolism, and energy production (Strasburg and Chiang, 2009; Malila et al., 2014). The presence of PSE-like meat still represents a large concern for vendors of fresh retail poultry cuts because of the moisture loss and abnormal color (Petracci et al., 2015), and also the value-added processing industry sector experiences great economic losses because of reduced protein functionality of affected meat (Owens et al., 2000; Barbut et al., 2008). For such reasons, research is encouraged to further investigate the effect of genotype, antemortem stressors, and slaughtering phases on the breast muscle acidification pattern of modern turkey hybrids to find solutions that can counteract the negative impact of PSE-like meat. A possible practical strategy could be to exploit abnormal meat for further processing through the addition of functional ingredients (i.e., phosphates, starches, hydrocolloids, vegetable proteins) to improve its technological properties (Barbut, 2009; Petracci et al., 2013).

In addition to PSE-like condition, dark-firm-dry (DFD) abnormality is well known by many years, and almost all farmed species show a certain prevalence of this quality defect affecting color and texture with detrimental effects on consumer willingness to purchase (Mallia et al., 2000; Fraqueza et al., 2006; Carvalho et al., 2018; Table 1). Also in turkeys, DFD results from an abnormal reduced postmortem muscle acidification (ultimate pH > 6.0) often associated with chronic or long-term stress experienced by the birds before slaughter (Barbut et al., 2008). Indeed, prolonged feed withdrawal and transportation, as well as exposition to cold temperature, can cause exhaustion of muscle glycogen stores and therefore a reduced postmortem lactic acid production through anaerobic glycolysis (Mallia et al., 2000; Fraqueza et al., 2006; Henrikson et al., 2018). DFD meat, being characterized by a darker-than-normal color and dry appearance, has a limited consumer appeal and thus is usually downgraded and redirected for further processing in turkey plants. Moreover, according to Fraqueza et al. (2006), darker turkey breast meat with higher ultimate pH values exhibits a fast microbial spoilage. Avoidance of long fasting period before slaughter and exposition to cold temperatures are well-known strategies to counteract DFD condition also in turkeys (Barbut et al., 2008; Henrikson et al., 2018).

During the 1980s, some meat quality issues related to the cohesiveness and juiciness of processed turkey breast meat, as well as to an overall toughening of the pectoralis major, were associated with microscopic, degenerative alterations of skeletal muscles in market turkeys, likely induced by the selection for growth rate and muscle yield (Dutson and Carter, 1985; Grey et al., 1986; Seemann et al., 1986; Grey, 1989; Sosnicki and Wilson, 1991). In a series of studies, Sosnicki et al. (1988a, b, 1989a, 1989b, 1991a, 1991b) investigated the pathology of skeletal muscles in fast-growing turkeys. The main degenerative alterations observed in the pectoralis major and iliofibularis (biceps femoris) muscle included scattered and focal necrosis, granular necrosis with phagocytosis by mononuclear cells of several adjacent muscle fibers or multifiber areas, hypercontraction of muscle fibers, as well as a strong proliferation of connective and fat tissues in the endomysium and perimysium (Sosnicki et al., 1991b). Sosnicki and Wilson (1991) defined this condition as the “focal myopathy” of the turkey. It is interesting to note that this myopathy was thought to be related with vascular defects, which could induce a localized microischemia at the terminal capillary bed level. Swatland (1990) observed a 35-fold increase in cross-sectional area of the muscle fibers of turkeys during the first 15 weeks of life, which was relatively greater than the growth of endomysial and perimysial connective tissue. Consequently, low values of capillary density and capillary-to-fiber ratio, as well as a greater intercapillary distance, were observed in necrotic regions of the pectoralis major and biceps femoris muscle of turkeys (Sosnicki et al., 1991a). Overall, these findings seem to indicate that the massive hypertrophic-based growth of muscle fibers was not accompanied by a concomitant growth of the connective layers, resulting in a poor vascularization of muscle fibers and then in histological defects and meat quality issues. Sosnicki and Wilson (1991) concluded that the physiological development of tissues supporting muscle as an organ (vascular, connective, adipose, nerve, etc.) has not kept pace with rapid growth and development of muscle fibers.

Intriguingly, the histological alterations observed almost 30 years ago in breast muscle of turkeys selected for high growth rate and meat yield are similar to those reported for breast muscle of fast-growing broiler chickens, which are affected by the emerging growth-related muscle abnormalities such as white striping (WS), wooden breast (WB), and spaghetti meat (SM) (Kuttappan et al., 2009, 2016; Petracci et al., 2014, 2015, 2019; Sihvo et al., 2014). Briefly, the WS condition is macroscopically characterized by the presence of white striations of variable thickness parallel to the direction of muscle fibers (Kuttappan et al., 2009), whereas WB one presents bulging and pale areas of muscle with hardened consistency (Sihvo et al., 2014). Recently, Baldi et al. (2018) described the SM condition as the tendency toward separation of muscle fiber bundles that mainly affects the superficial layer of the cranial region of pectoralis major muscle. As reviewed by Soglia et al. (2019), all these abnormalities share some common microscopic features such as the loss of normal muscle architecture, presence of abnormal fibers presenting rounded profiles, nuclear internalization, fiber degeneration, necrosis up-to-lysis associated with concurrent regenerative processes (small-caliber regenerative fibers intermingled to abnormal ones having large caliber), compromised perimysial and endomysial connective tissue, and fat and inflammatory cell infiltrations. In addition, each syndrome also exhibits distinctive histological traits such as an abnormal deposition of adipose tissue in the perimysium (lipidosis) for WS and proliferation and thickening of the perimysial network (fibrosis) in WB condition. In contrast, the SM defect is characterized by a progressive rarefaction of the endomysial and perimysial connective tissue layers (Soglia et al., 2019).

In turkeys, the presence of white striations in the superficial layer of pectoralis major muscle, as well as the tendency of muscle fiber bundles to separate, has been observed in commercial practices especially in heavy male turkey hybrids but not considered a main quality issue by producers or consumers. Past investigations (Grey et al., 1986; Seemann et al., 1986; Grey, 1989) also described a turkey meat quality issue characterized by an increased toughness of the pectoralis major muscle coupled with the presence of giant hypercontracted cells, fiber defects, and overall greater muscle cell size (Sosnicki and Wilson, 1991). In addition, the hypothetical causative mechanism of emerging muscle abnormalities in broiler chickens (i.e., limited capillary density, impaired oxygen supply, and metabolic waste product displacement from breast myofibers) seems to be analogous to that hypothesized by Sosnicki and Wilson (1991) for turkeys. However, in contrast to broiler chickens, there is limited information regarding emerging growth-related muscle defects in modern turkeys. In addition, considering the detrimental effects of these muscle abnormalities on meat quality traits in chickens, it is fundamental to understand whether this scenario could be similar for turkeys and also if the genotype could have any impact on the occurrence of such conditions.

## Occurrence of Breast Meat Abnormalities in Modern Turkey Hybrids

In broilers, the genetic potential for breast meat yield and growth rate is considered the main factor affecting the occurrence of breast abnormalities as recently reviewed by Petracci et al. (2019). Previous studies on turkeys showed that the incidence of meat abnormalities and muscle fiber defects was greater in hybrids selected for high growth rate and breast muscle development (Wilson et al., 1990; Sosnicki and Wilson, 1991; Velleman et al., 2003). However, the effects of these promoting factors on turkey meat quality are somewhat less clear.

To examine the effects of growth selection on turkey meat quality, Updike et al. (2005) compared breast meat functionality in three lines of turkeys of differing growth traits: (1) a randombred control line (RBC2), representing commercial turkeys of the 1960s; (2) F line, which was selectively bred from RBC2 line for increased body weight at 16 weeks; and (3) a fast-growing commercial line. The RBC2 turkey breast meat showed highest water-holding capacity and tenderness (measured by Warner-Bratzler shear force) compared to that of the other two lines. Although there were no differences in cook yield, thermally induced meat gels from the RBC2 line were strongest of the three lines. Overall, these results support the hypothesis that growth selection can negatively affect meat quality.

Environmental conditions such as heat or transportation stress may differentially affect meat quality of slow-growing and fast-growing turkeys. Chiang et al. (2008) subjected RBC2 and commercial turkeys to various periods of heat stress and examined differences in meat quality and thyroid hormone (T3 and T4) levels. They observed that the breast muscle from fast-growing commercial turkeys had higher pH at 15 min postmortem and greater cook yield compared to the slow-growing randombred line, whereas no differences were detected in water-holding capacity. However, of particular note from a processed meat standpoint is the observation that meat from the RBC2 line had a significantly higher marinade uptake than that of commercial birds. Moreover, this parameter was very consistent over the first 3 days of heat stress, whereas that of the commercial line fluctuated significantly as a function of time. Marinade uptake is an important indicator of protein functionality, and higher ability to pick up marinade solutions is generally associated with greater quality meat products. Interestingly, the changes in marinade uptake in the commercial line were mirrored by changes in the plasma T3 levels. Both marinade uptake and T3 levels were much more consistent in the RBC2 line. Taken together, this study also supports the hypothesis that selection for growth performance adversely affects some meat quality parameters, particularly upon environmental stress. On the other hand, Werner et al. (2008) analyzed breast meat from two fast-growing and two slow-growing turkey lines and found no clear differences among the tested turkey strains. One of the fast-growing lines showed a significantly higher drip loss compared to the others, and muscle from both fast growing lines exhibited higher fibers diameter, although the ratio of capillaries to unit of fiber area was not different between lines. It is important to note that although the birds in this study were raised and harvested simultaneously under the same conditions, they were not subjected to environmental stress. Thus, the effects of growth selection on the PSE-like condition in turkeys may depend on environmental effects.

Whereas DPM and PSE-like conditions have been extensively studied in the past, there is a lack of scientific information about other potential emerging growth-related abnormalities in turkeys. To address these concerns, a trial was carried out by our research group to evaluate the occurrence of WS, WB, and SM-like abnormalities in two commercial turkey hybrids (*n* = 486 toms per genotype A and B, respectively) (Zampiga et al., 2019). The genetic lines tested in the trial are currently available and used worldwide for turkey meat production. The lines belong to two major independent breeding companies, were not genetically related, and were fed the same diet and raised in the same environmental conditions. At 140 days, the toms, which showed comparable growth performance at the end of the trial, were processed in a commercial slaughterhouse following the procedure commonly adopted in the EU. The occurrence of breast meat abnormalities was assessed on 100 breasts/genotype ˜24 h after processing. To evaluate the severity of the WS defect, a 4-point scale evaluation system was developed: 0 = no lesions; 1 = mild lesions: few stripes; 2 = marked lesions: up to 50% of the breast surface covered by the white striations; 3 = severe lesions: more than 50% of the breast surface covered by the white striation (Zampiga et al., 2019). For the WB and SM-like defect, the evaluation systems proposed by Sihvo et al. (2014) and Sirri et al. (2016) for broiler chickens were used, respectively. The percentage of breasts affected by WS was markedly higher in both the tested lines as indicated by the very low percentage (1%) of breasts reporting no WS lesions. Moreover, a significant effect of the genotype was observed on the frequency of the different severity classes. Turkeys from group A showed a significantly lower percentage of breasts presenting severe lesions (46 vs. 60%, respectively, for A and B) and a higher incidence of those with mild lesions (17 vs. 5%, respectively, for A and B). On the other hand, the overall occurrence of WB and SM-like conditions was negligible, and no significant difference emerged between the genotypes. The low incidence of the SM-like defect in breast muscle of turkeys might be partially explained by the analysis carried out to assess the intramuscular collagen properties in breasts presenting no macroscopic defects. Overall, the two turkey strains showed a similar breast muscle collagen content and, regardless of the genotype, were characterized by a good level of collagen cross-linking and maturation. These findings could support the hypothesis of a proper muscle structure, which could explain the overall low occurrence and severity of SM-like condition in both the genetic lines (Zampiga et al., 2019). Mudalal (2019), analyzing 2,300 turkey breasts from 22 flocks (20 weeks old turkeys), found that the overall occurrence of moderate and severe WS was 61.3%, with moderate cases accounting for 49.4%. Moreover, it has been recently reported that a visible-near infrared spectroscopy-based approach was useful to differentiate normal from severe WS turkey fillets, which could have a potential application in the turkey industry (Zaid et al., 2020).

Zampiga et al. (2019) also performed histological evaluations on pectoralis major muscles representative of each abnormality and degree of severity adopted for testing the occurrence of muscle myopathies (i.e., WS = 0, 1, 2, and 3; and SM = 0 and 1). Breast muscles not showing WS defect (WS = 0) exhibited myofibers with a regular polygonal profile, as well as endomysial and perimysial connective tissues without remarkable alterations. In breast muscles macroscopically presenting minor WS lesions (WS = 1), some necrotic fibers intermingled with apparently normal ones that have lost their polygonal profile, as well as changes of the perimysial connective layer, were observed. In severe WS (WS = 2 and 3), histological sections exhibited nuclear internalization, vacuolar and hyaline degeneration, necrosis and lysis of the fibers, inflammatory cell infiltration, variable fiber cross-sectional area (degenerating and regenerating fibers), lipidosis, and fibrosis. A similar scenario, including degenerative changes such as scattered and focal necrosis, hypercontraction of muscle fibers, and strong proliferation of connective and fat tissues in the endomysium and perimysium, as well as infiltration of the necrotic areas by mononuclear cells, has been previously reported in pectoralis major muscle of fast-growing turkeys (Sosnicki et al., 1988a, b; Sosnicki and Wilson, 1991). Overall, these histological findings are consistent with the muscle alterations reported in previous studies conducted on commercial broiler chickens (Kuttappan et al., 2013; Radaelli et al., 2017; Baldi et al., 2018).

On the other hand, breasts affected by the SM-like defect showed a distinctive progressive rarefaction of the endomysial and perimysial connective tissue, leading to muscle fibers detaching from each other. This compromised connective tissue was generally accompanied by degenerate and necrotic (up-to-lysis) fibers and fat and inflammatory cells infiltration, as well as the presence of small-caliber fibers associated with large-caliber ones showing rounded profile. To the best of our knowledge, the data reported in Zampiga et al. (2019) are the only ones available in literature regarding the SM-like condition in turkeys. Sosnicki and Wilson (1991), discussing the focal myopathies, pointed out that the excrescence of the sinus muscle fibers on the supporting connective tissue may also predispose the products to fragmentation, which could be similar to the recently observed SM-like condition. Moreover, these findings are in agreement with those previously reported by Baldi et al. (2018) regarding histological sections of broiler breast muscle affected by the SM abnormality.

Taken together, the overall occurrence of WS in commercial turkey hybrids was remarkably high, whereas that of SM-like and WB was sporadic and negligible, respectively (Table 1). Nonetheless, it should be considered that, differently from broiler meat, the presence of white striations in turkey breasts seems not to significantly affect consumers’ perception possibly because most of the turkey meat is commercialized worldwide as ready-to-eat products and meal (Remignon, 2004). Similarly, other marketing forms such as manufacturing sliced (unprocessed, raw slices) or whole carcass (e.g., for Thanksgiving and Christmas festivity meals), which are common, respectively, in the EU and US market, tend to reduce the impact of the white striations typical of the WS defect. Finally, the histological alterations found in turkey breast muscles presenting WS and SM-like conditions were similar to those reported for broilers. However, the overall degree of severity was remarkably lower in turkeys, with possible implications on overall meat quality traits that will be discussed in the following section of this review.

## Effect of Ws on Proximate Composition and Technological Traits of Breast Meat

In chickens, breast muscle abnormalities have been associated with marked alterations in meat chemical composition and technological traits as recently reviewed by Petracci et al. (2019). In general, WS, WB, and SM conditions are characterized by increased amount of fat and moisture and lower protein content compared to unaffected breasts (Petracci et al., 2014, 2019; Baldi et al., 2018). In addition, WB- and WS-affected breasts displayed impairment of meat functionality resulting in poor technological traits, particularly for WB condition (Tijare et al., 2016), which could have a tremendous impact on processing attitude and hence on overall quality features of value-added processed meat products that represent an important form of commercialization for turkey meat (Remignon, 2004).

The effects of the WS abnormality on proximate composition and technological traits of turkey breast meat were recently investigated by Soglia et al. (2018). As reported by the authors, a total of 72 boneless and skinless pectoralis major muscles were collected 48 h postmortem and classified according to the severity of WS (i.e., thickness of the white striations in the ventral surface of the pectoral muscles) as normal (NORM), moderate (MOD), and severe (SEV). All the breasts were obtained from two separate trials on two flocks of heavy male turkeys (BUT Big 6). Rearing, feeding, and slaughtering conditions were representative of current commercial practices in the EU. Technological traits, such as pH, drip loss, cooking loss, tenderness, marinade uptake, and nuclear magnetic resonance (NMR) relaxation properties, as well as proximate composition, were determined.

Overall, fillets presenting the WS defect were characterized by a significantly greater weight (˜20%) compared to the normal ones, potentially confirming a growth-related origin of WS also for turkeys (Soglia et al., 2018). Similarly, previous investigations hypothesized an association between muscle degeneration and growth rate of the turkeys, with the ones showing rapid growth rate being characterized by severe degenerations (Wilson et al., 1990; Velleman et al., 2003).

However, unlike broiler chickens, the presence of WS in turkeys had a limited effect on proximate composition. Indeed, breasts presenting WS showed no significant difference in moisture and protein content, whereas total lipid was higher in SEV group compared to the NORM counterpart. Moreover, regardless of the severity of WS abnormality, affected breasts exhibited lower ash content in comparison with NORM ones. No significant differences were observed for collagen content among the groups. Mudalal (2019) reported that turkey breasts presenting severe WS were characterized by higher fat and lower protein content as well as different color indexes (a^∗^ and b^∗^) compared to unaffected ones. Overall, these findings corroborate with the results of previous studies performed on broilers showing WS (Kuttappan et al., 2013; Petracci et al., 2014). Indeed, the differences in proximate composition can be explained by considering the degenerative and regenerative processes taking place within these muscles, which involve the proliferation of connective tissue and fat infiltration as microscopically observed in the skeletal muscle tissue of turkeys showing WS abnormality (Zampiga et al., 2019).

Consistent with the limited differences found in proximate composition, also the technological traits were slightly affected by the presence of WS abnormality (Soglia et al., 2018). Indeed, with the only exception of drip loss that significantly differed among the groups, no remarkable alterations were observed for overall quality traits of both raw (pH, color, cooking losses, and shear force) and marinated (uptake, cooking losses, and shear force) meat. The lack of differences in water-holding capacity using conventional drip loss determination was confirmed by the NMR analysis aimed at assessing the water mobility and distribution within the muscle tissue. Therefore, in contrast to the studies reported for broilers, an overall absence of adverse effects of WS on breast meat quality traits of turkey was observed. The authors supposed that this difference could be explained by species-specific dissimilarity in breast muscle morphology and development among turkeys and broilers. Indeed, the increase in breast meat yield obtained by selection process in broilers was mainly accomplished by enhancing breast muscle thickness (Soglia et al., 2018). However, it was reported that the greater the pectoralis major depth and yield, the higher the likelihood to find severe myopathic alternations within the muscle tissue (Griffin et al., 2018). Therefore, it has been hypothesized that the limited impact of WS on turkey meat quality might be ascribed to a more harmonious development of breast muscle that, being not merely restricted to the depth as observed in broilers, could lead to less pronounced myopathic changes within the muscle tissue (Soglia et al., 2018).

Taken together, differently from broiler chickens, the overall impact of emerging growth-related breast muscle myopathies (WS and SM) in turkey seems rather limited. On the other hand, the presence of PSE-like meat is still a major concern for this sector (Table 1). Based on the findings obtained in previous studies carried out on broilers, several feeding strategies can be implemented with the aim to reduce the incidence of these growth-related abnormalities as extensively reviewed by Petracci et al. (2019). However, the same authors suggested that a reduction in the incidence of myopathies in response to feeding strategies might be ascribed to an overall limitation of muscle development and growth performance rather than to a real effect associated with the dietary treatments.

## Conclusion

The overall occurrence of WS in commercial turkey hybrids was remarkably high, whereas that of SM-like and WB conditions could be considered sporadic and negligible, respectively. Moreover, a possible growth-related origin for WS in turkeys could be assumed. However, despite its high occurrence and the fact that histological alterations are similar to those reported for broilers, WS condition did not significantly affect meat quality traits in turkeys resulting in no negative impact either for raw or processed meat products. Taken together, unlike broiler chickens, growth-related breast muscle abnormalities are not a main concern at present for the turkey meat production chain. In this scenario, the PSE-like condition or the proneness of the meat toward oxidation, both during refrigerated and frozen storage, could be considered the main quality issues, and hence other insights are necessary to find practical solutions which could alleviate these problems.

## Author Contributions

MZ wrote the first draft of the manuscript. All authors contributed to the manuscript revision, and read and approved the submitted version.

## Conflict of Interest

The authors declare that the research was conducted in the absence of any commercial or financial relationships that could be construed as a potential conflict of interest.
